# Median Nerve Lipofibrohamartoma: A Treatment Dilemma

**Published:** 2017-09

**Authors:** Bharat Mishra, Jerry R. John, Satyaswarup Tripathy, Ramesh Kumar Sharma

**Affiliations:** Department of Plastic Surgery, Panjab University, PGIMER, Chandigarh, India

**Keywords:** Median nerve, Lipofibrohamartoma, Carpal tunnel, Biopsy

**DEAR EDITOR**

Carpal tunnel syndrome is a commonly encountered entity. The tunnel is a narrow space filled with nerves and tendons. Numbness and paraesthesia over the median nerve territory is classical of nerve compression. Pregnancy, hypothyroidism and diabetes are frequent associations, but sometimes no cause can be ascertained. Local pathologies like ganglia and other soft tissue tumors occasionally result in nerve compression.^1^

A middle aged lady presented with numbness over the thumb, index and middle fingers over her hand. These complaints were present for nearly a year and were more severe in the mornings. She had no history of comorbidities. On examination, there was an ill-defined bulge over her wrist. No definite swelling was palpable. No thenar wasting was present and no sensory weakness was detected. Phalen’s test was positive. The contralateral hand was asymptomatic. A provisional diagnosis of carpal tunnel syndrome was made. The cause, however, was not apparent. 

An MRI was ordered to further clarify the cause of fullness of the wrist. This revealed a diffuse enlargement of the median nerve with a ‘cable-like’ pattern (Figure 1), at the region of the wrist and extending into the palm.^2^ A coronal section showed the ‘spaghetti sign’, suggesting the cause to be lipofibrohamartoma of the median nerve. The patient was counselled regarding the nature of the disease. She opted for surgical decompression of carpal tunnel and a diagnostic biopsy of the lesion. 

**Fig. 1:**
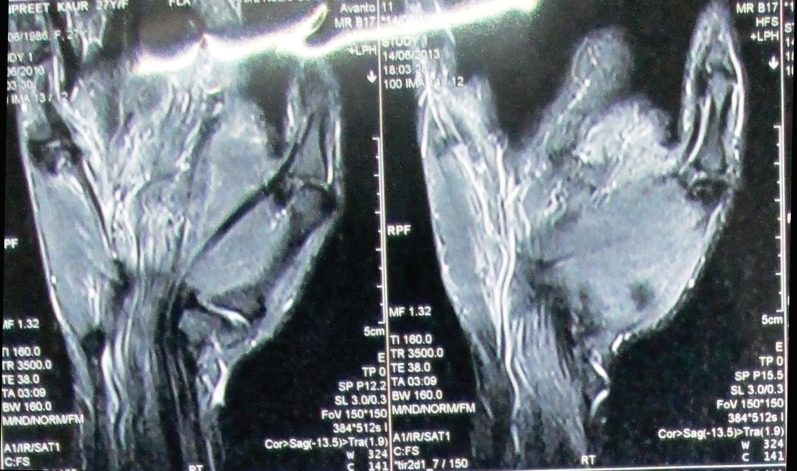
MRI picture shows the pathognomonic ‘cable-like’ pattern of the nerve

The carpal tunnel was released by the open method using an exploratory incision on the wrist extending to the mid-palm. The median nerve fibers appeared larger than usual and were intricately encased in glistening yellow fibrofatty tissue (Figure 2). The epineurium was then split open. The fatty component was teased away from the neural elements with the idea of obtaining sufficient tissue for biopsy. Excision or debulking of this fatty component without causing nerve damage was not possible. Histopathological examination showed fatty infiltration and fibrosis of the epiperineurial tissues.^3^ Immunohistochemistry demonstrated that the tissue was negative for S-100 (Figure 3).

**Fig. 2:**
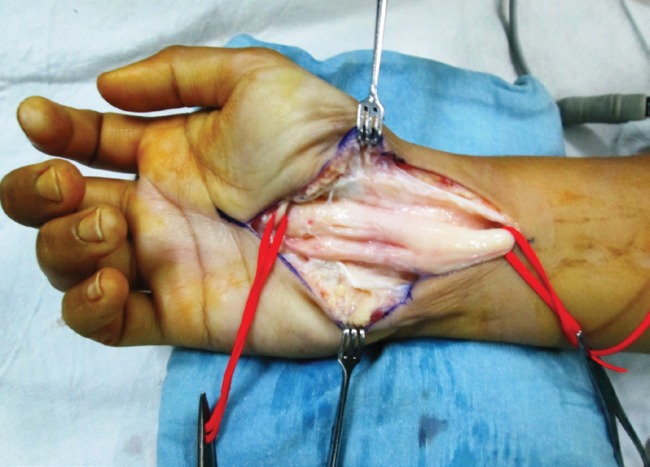
Intraoperative view of the enlarged nerve

**Fig. 3:**
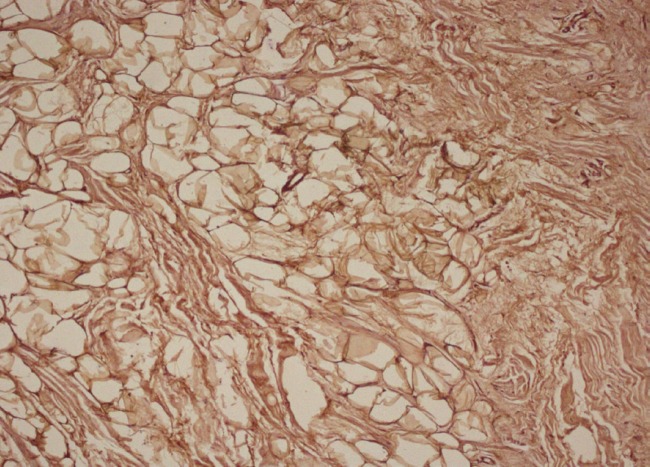
Histopathological examination showed fibrofatty infiltration of the epiperineurium

In carpal tunnel syndrome, median nerve is most often ‘a victim of circumstances’. In this case however, it is ‘the culprit’. Primary abnormality of the median nerve causing carpal tunnel syndrome is rare. Local wrist fullness prompted us to order an MRI, which revealed the pathology. This helped in (a) ascertaining the cause, (b) patient education prior to surgery and (c) preventing an intraoperative surprise. Lipofibrohamartoma of the median nerve is a benign disease. It presents most often in children and young adults. Complaints most often suggest carpal tunnel syndrome, but paresis of the median nerve is also possible. An association with macrodactyly has been postulated.^4^

Due to paucity of cases, no standard guidelines exist for management of this pathology. The surgeon is confronted with the dilemma of deciding how extensive his surgery should be. That the carpal tunnel should be opened up is obvious. To confirm and assess the extent of the disease, we chose the open method of carpal tunnel release. Since the fibrofatty tissue is inextricably interwoven with the nerve fibers, excision or debulking was not possible. The amount of this tissue to be removed must be decided intraoperatively. We chose to do a biopsy alone, since this minimised the possibility of nerve damage. Others have proceeded further, with neurolysis and debulking and even segmental excision and grafting.^5^

In conclusion, it is worthwhile to watch out for rare causes of common complaints. Meticulous clinical examination and appropriate preoperative tests can prevent intraoperative surprises. MRI findings are reliable enough to make a provisional diagnosis of lipofibrohamartoma. The surgeon must decide how aggressive to be, in operating such a rare pathology. Release of the carpal tunnel and diagnostic biopsy can be performed with no morbidity. 

## CONFLICT OF INTEREST

The authors declare no conflict of interest.
